# The collaborative multi-level lot-sizing problem with cost synergies

**DOI:** 10.1080/00207543.2019.1584415

**Published:** 2019-03-04

**Authors:** Margaretha Gansterer, Richard F. Hartl

**Affiliations:** Department of Business Decisions and Analytics, University of Vienna, Oskar-Morgenstern-Platz 1, 1090 Vienna, Austria

**Keywords:** lot-sizing, decentralised planning, genetic algorithms

## Abstract

Collaborative operations planning is a key element of modern supply chains. We introduce the collaborative multi-level lot-sizing problem with cost synergies. This arises if producers can realise reductions of their costs by providing more than one product in a specific time horizon. Since producers are typically not willing to reveal critical information, we propose a decentralised mechanism, where producers do not have to reveal their individual items costs. Additionally, a Genetic Algorithms-based centralised approach is developed, which we use for benchmarking. Our study shows that this approach comes very close to the a central plan, while in the decentralised one no critical information has to be shared. We compare the results to a myopic upstream planning approach, and show that these results are almost 12% worse than the centralised ones. All solution approaches are assessed on available test instances for problems without cost synergies. For the biggest available instances, the proposed centralised mechanism improves the best known solutions on average by 10.8%. The proposed decentralised mechanism can be applied to other problem classes, where collaborative decision makers aim for good plans under incomplete information.

## 1. Introduction

Firms acting in competitive environments are forced to collaborate with other players within their supply chain networks in order to overcome inefficiencies and to stay in business. This can be done by coordination or collaborative planning, which are key elements of supply chain management. Various coordination schemes have been presented in the literature (e.g. Xu et al. 2014). However, collaborative planning is more than just a coordination of plans. It is defined by Stadtler (2009) as individual plans that are adapted in an effort of joint decision-making, i.e. a willingness to cooperate and to contribute to the generation of a plan which will be accepted by the collaborating players. Thus, collaborative planning is neither pure information sharing, nor centralised planning, where one actor (which might be a central authority) possesses full information. However, collaborative planning might require that actors are willing to reveal parts of their private information to their competitors.

Digitalisation and Industry 4.0 make it possible that data can be exchanged extremely fast and in secure environments. However, firms are still not willing to reveal critical data like their existing customers, cost structure or capacities. Thus, collaborative planning demands for distributed decision-making mechanisms, where no sensitive information has to be shared. Cloud Manufacturing is an emerging concept that enables collaboration in the context of manufacturing, in which systematic orchestration, matching, and sharing of services and components are the key elements (Moghaddam and Nof 2018; Chen, Fang, and Tang 2019; Wang et al. 2019). Several real-world cases have recently been discussed in literature, e.g. Leng and Jiang (2018).

Multi-level lot-sizing is a major driver of the costs and customer service in supply chain networks. Lee and Kumara (2007a) claim that because of its practical importance it is among the most widely researched areas in operational supply chain management (e.g. Tempelmeier and Helber 1994; Tempelmeier and Derstroff 1996; Dellaert and Jeunet 2000; Pitakaso et al. 2007; Buschkühl et al. 2010; Wei et al. 2019). Several decentralised collaboration mechanisms for distributed multi-level lot-sizing problems have been presented in the literature (e.g. Lee and Kumara 2007a; Homberger 2010; Buer 2013). All proposed mechanisms are not dedicated to settings, where the assignment of items to producers bears some flexibility. However, today's highly complex supply chain networks are based on modularisation and service-orientation. Hence, they consist of various flexibly operating producers and settings where some items could be produced by more than one potential supplier (Moghaddam and Nof 2018). As a matter of fact, Buer, Ziebuhr, and Kopfer (2015) introduced the collaborative lot-sizing problem with rivalling agents. A negotiation-based approach is proposed to find beneficial assignments of items to agents as well as profitable production plans.

In practical applications, agents will be assigned with more than one item. Production of several products is known to come with mutual cost synergies due to joint setups (Jans and Degraeve 2008), replenishments (Federgruen and Tzur 1994; Hezarkhani, Slikker, and Van Woensel 2018), or transportation (Ke and Bookbinder 2018). While the multi-level lot-sizing problem is known to be NP-hard (Homberger 2010), the extension to rivalling agents clearly increases complexity. In our study we introduce cost synergies, meaning that producing a set of items has less total cost than the sum of the costs of each item produced individually. The exploration of the solution space gets even harder if agents are facing cost synergies, since each assignment has a direct influence on the decision about all other items that might be produced by the same agent (an illustrative example is provided in Section 3).

While cost synergies seem to be a natural and highly practical extension, they have not been considered in the literature on distributed multi-level lot-sizing problems so far. Our study is closing this research gap and delivers the following contributions:

- We are the first to introduce and mathematically formulate the centralised collaborative multi-level lot-sizing problem with cost synergies, which is a non-linear problem. A Genetic Algorithm (GA) is developed to solve this problem.
- We propose a decentralised mechanism to tackle the problem, where agents do not have to share sensitive information. It includes a GA-based approach to produce production plans. This mechanism is benchmarked against centralised solutions generated by a fully informed decision maker. We show that the decentralised mechanism, although no critical information is shared, comes very close to the centralised solutions.
- We show the value of collaborative planning by comparing a myopic planning approach against the proposed decentralised mechanism.
- All solution approaches are assessed on available test instances for problems without cost synergies. This emphasises the strength of the proposed decentralised mechanism.
- This proposed mechanism can also be applied to related problems, where collaborative decision makers aim for good plans under incomplete information (e.g. collaborative replenishment or distribution).

The remainder of the paper is organised as follows. Section 2 provides a literature review. Details on the problem definition are given in Section 3. We design the solution method in Section 4. The computational study is presented in Sections 5 and 6 concludes.

## 2. Literature review

In our literature review we bring together two streams of research, which are the one on operations pooling and on distributed decision-making. Various collaborative planning schemes have been introduced in the literature (Fransoo, Wouters, and de Kok 2001; Taghipour and Frayret 2013; Thomas et al. 2015; Gansterer and Hartl 2018b; Pan et al. 2019). These are referred to as *horizontal*, if participants act at the same levels in a market (Cruijssen, Dullaert, and Fleuren 2007). Vertical cooperations on the other hand, indicate hierarchical relationships, meaning that one player is the client of the other. In Schneeweiss (2003) different hierarchical relationships in distributed decision-making are elaborated.

Typically, these mechanisms help participants to orchestrate their production plans but they do not consider shared resources or an exchange of customer orders. For instance, Lai, Cai, and Li (2017) consider firms making collaborative production-distribution planning with shipment consolidation to reduce costs. The authors develop a computable mechanism based on a decentralised local search heuristic combined with simulated annealing. A decentralised production-distribution planning system using collaborative agents is proposed by Jung and Jeong (2005). A survey of factory control algorithms that can be implemented in a multi-agent heterarchy is presented by Baker (1998). A coordination problem in a two-level assembly system with stochastic lead times is researched by Tang and Grubbström (2003). Chen et al. (2019) propose a cooperative approach to service booking and scheduling in cloud manufacturing.

Most of the studies explicitly dealing with pooling of operations, assume a central decision maker having full information. Drechsel and Kimms (2011) study the cooperative capacitated lot-sizing problem where the available resources of the players may be used in common. Lamas and Chevalier (2013) contribute to the issue of fairness in operations pooling in the absence of transfer payments. A new class of cooperative games that arise from production-inventory problems, where several agents share production processes and warehouse facilities is presented by Guardiola, Meca, and Puerto (2009). A path towards resource efficiency and optimal material usage in manufacturing is researched by Aminoff and Paajanen (2018). A real-world case of a collaborative manufacturing network of a printing machinery is researched by Leng and Jiang (2018). However, these studies do not cover decentralised planning mechanisms, where players are reluctant to share information.

Collaborative agents competing on tasks or orders are basically present in the field of production scheduling but not for lot-sizing decisions. Kutanoglu and Wu (2006), for instance, present an auction-based mechanism for the collaborative production scheduling problem that arises when schedulers must coordinate their schedules with internal or external customers. Resource allocation is communicated in the form of schedules over which each agent involved has different preferences and financial incentives. A multi-agent framework for the coordination and integration of information systems is presented by Sikora and Shaw (1998). The framework is applied to the development of a manufacturing information system for managing the production processes for making printed circuit boards.

Literature on distributed lot-sizing problems, which we investigate in our study, is scarce. Related early studies include Monahan (1984) and Lee and Rosenblatt (1986). Lee and Kumara (2007a) design a decentralised coordination mechanism for dynamic lot-sizing in distribution networks. Dudek and Stadtler (2005) investigate negotiation-based collaborative planning between supply chains partners. A multi-agent system approach for dynamic lot-sizing in supply chains is discussed in Lee and Kumara (2007b). The authors claim that there is a need to design trustworthy mechanisms, where agents are guaranteed to get the right benefits in return for information sharing. Zoghlami et al. (2016) research the management of divergent production networks using decentralised multi-level capacitated lot-sizing models. The decentralised multi-level uncapacitated lot-sizing problem is firstly presented by Homberger (2010). A collaborative ant colony metaheuristic for the same problem is developed by Buer (2013). A generic mechanism to coordinate decentral planning of a group of independent and self-interested decision makers, who are searching for an agreeable contract regarding multiple interdependent issues, in the case of asymmetric information is presented by Homberger (2011). Shapley-based side payments and simulated annealing for distributed lot-sizing are researched by Eslikizi et al. (2015). Buer, Ziebuhr, and Kopfer (2015) are the first to present the collaborative lot-sizing problem with rivalling agents, where the assignment of items to agents is flexible. The authors propose a negotiation approach based on simulated annealing. The assignment of items to agents is done using the total item costs per agent. The same problem including production limitations is investigated in Ziebuhr, Buer, and Kopfer (2015).

To the best of our knowledge, cost synergies in distributed lot-sizing problems have not been researched so far. However, this is a natural extension, since synergies are the main motivation for any kind of collaboration (see Section 1).

## 3. Problem description

In our study we present the decentralised multi-level lot-sizing problem with cost synergies. It is based on the decentralised multi-level uncapacitated lot-sizing problem firstly presented by Homberger (2010), and further investigated by Buer (2013), and Buer, Ziebuhr, and Kopfer (2015). Buer, Ziebuhr, and Kopfer (2015) are the first to tackle the problem with rivalling agents, where the assignment of items to agents is flexible. In line with Buer, Ziebuhr, and Kopfer (2015) we assume that some parts can only be produced by a specific agent (*compulsory items*), while others have more than one potential producer (*concurrent items*). Additionally, we assume that producers face cost synergies, meaning that producing a set of items has less total cost than the sum of the costs of each item produced individually. This is a natural extension, since all kinds of collaborations are motivated by such synergy effects. In the problem at hand, such synergies might occur due to joint setups (Jans and Degraeve 2008), replenishments (Federgruen and Tzur 1994; Hezarkhani, Slikker, and Van Woensel 2018), or transportation (Ke and Bookbinder 2018). Thus, a single item might be too costly for a producer, while it is attractive when offered in a bundle with other items.

**Figure 1.**
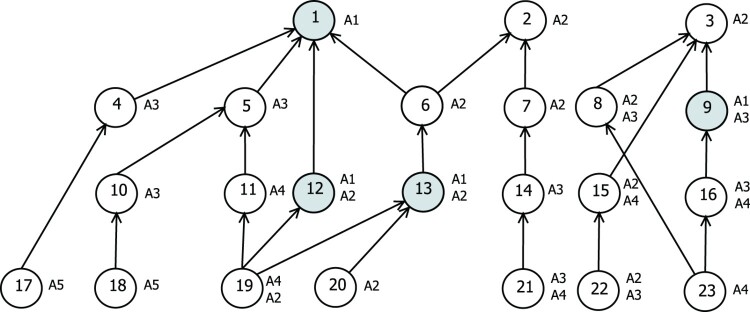
Illustration of a product structure with 24 items being produced by 5 agents (A1–A5). Compulsory items (e.g item 1) and concurrent items (e.g. item 8) are illustrated. It is assumed that agents can benefit of cost synergies if they produce bundles of items that are on the same BOM level or if they are directly connected within the product structure.

We assume that both the assignment of items to agents as well as the lot-sizing decision is done once within one planning interval. We illustrate the problem in Figure 1 using a bill-of-materials (BOM), i.e. the product structure including all end products and their components. For this, we assume that three end products are produced by two agents (A1, A2). There are four bill-of-materials levels, where five agents are involved (A1,…,A5). Most of the products are concurrent items, meaning that more than one agent is able to produce it. Let us assume cost synergies for items being on the same BOM level, and for items where one is the component of the other one. Depending on the costs of the other agents, it might be beneficial to let agent 1 not only produce item 1 but also items 12 and 13. This bundle of items might give agent 1 a cost advantage, since items 12 and 13 are on the same BOM level, and item 12 is directly connected to item 1. It seems that item 9, on the other hand, does not fit into this bundle. It might be more profitable to bundle this one with items 8 and 16 and assign them to agent 3. However, we assume that there is no central authority having access to individual costs of the agents. Thus, we propose an algorithm that is able to find profitable assignments under incomplete information. It should be noted that this assignment has a major impact on the production plans of all other items as well. Different agents might have different cost structures and therefore different production plans.

The multi-level lot-sizing problem with cost synergies, or more general the multi-level lot-sizing problem with rivalling agents, occurs in highly interlaced supply chain networks of flexible manufacturers. For a better understanding of the underlying problem, we provide the mathematical formulation of the *centralised* multi-level lot-sizing problem with cost synergies, where we assume a central decision maker having full information. Note that for our decentralised solution approaches to be presented in Section 4, we will assume the more realistic case that no such fully informed decision maker exists. However, the proposed centralised planning approach optimises the following non-linear and integer problem. We use the following list of symbols.

$I_{1}$
set of compulsory items, $i\in I_{1}$
$I_{2}$
set of concurrent items, $i\in I_{2}$
*I*: set of all items, i∈I, I=1,…n, $I=I_{1}\cup I_{2}$
*A*: set of all agents, a∈A
$A_{i}$
set of agents being able to produce item *i*
*T*: set of periods, t∈T, T={0,…,τ}
Γ(i)
set of items being direct successors of item *i* in the product structure
$\Omega_{ia}$
binary parameter indicating whether item *i* is a compulsory item of agent *a*
*M*: sufficiently large number
$s_{ia}$
setup cost (in monetary units) of agent *a* producing item *i*
$h_{ia}$
unit holding cost of agent *a* for storing one entity of item *i*
$c_{ia}$
unit cost of item *i* being produced by agent *a*
$p_{ij}$
production coefficient giving the amount of item *i* needed to produce one entity of item *j*
$D_{it}$
exogenously given demand of product *i*
$\omega_{ija}$
factor by which setup cost $s_{ja}$ is reduced, if both, products *i* and *j* are assigned to agent *a*, i.e. $\omega_{ija}=0$ if there is no synergy effect and $\omega_{ija}=0.1$ if there is a 10% reduction because of synergy
${\bar{\omega }}_{ija}$
factor by which unit holding cost $h_{ja}$ is reduced, if both, products *i* and *j* are assigned to agent *a*
${\tilde{\omega }}_{ija}$
factor by which unit cost $c_{ja}$ is reduced, if both, products *i* and *j* are assigned to agent *a*
$x_{ia}$
binary decision variable indicating whether item *i* is assigned to agent *a*
$y_{iat}$
binary decision variable indicating whether agent *a* is producing item *i* in period *t* (setup)
$z_{ija}$
binary variable indicating that both, products *i* and *j* are assigned to agent *a*
$q_{it}$
quantity of item *i* being produced in period *t*
$I_{it}$
quantity of item *i* being stored at the end of period *t*
$d_{it}$
endogenous demand of item *i* in period *t*

(1) $$\begin{array}{rl} & \min\sum_{i\in I}\sum_{a\in A}\sum_{t\in T}\left[s_{ia}y_{iat}\prod_{j\in I\setminus \left\{i\right\}}(1-\omega_{jia})z_{ija}\right.\\ & \;+h_{ia}x_{ia}I_{it}\prod_{j\in I\setminus \left\{i\right\}}(1-{\bar{\omega }}_{jia})z_{ija}\\ & \;\left.+c_{ia}x_{ia}q_{it}\prod_{j\in I\setminus \left\{i\right\}}(1-{\tilde{\omega }}_{jia})z_{ija}\right], \end{array}$$

(2) $$\begin{array}{rl} & I_{it}=I_{it-1}+q_{it}-d_{it}-D_{it}\;\forall \;i\in I,t\in 1,\ldots ,\tau , \end{array}$$

(3) $$\begin{array}{rl} & I_{i0}=0\;\forall \;i\in I, \end{array}$$

(4) $$\begin{array}{rl} & d_{it}=\sum_{j\in \Gamma (i)}p_{ij}q_{jt}\;\forall \;i\in I,t\in T, \end{array}$$

(5) $$\begin{array}{rl} & q_{it}\leq M\sum_{a\in A_{i}}y_{iat}\;\forall \;i\in I,t\in T, \end{array}$$

(6) $$\begin{array}{rl} & y_{iat}\leq x_{ia}\;\forall \;i\in I,a\in A,t\in T, \end{array}$$

(7) $$\begin{array}{rl} & x_{ia}=\Omega_{ia}\;\forall \;i\in I_{1},a\in A, \end{array}$$

(8) $$\begin{array}{rl} & \sum_{a\in A_{i}}x_{ia}=1\;\forall \;i\in I_{2}, \end{array}$$

(9) $$\begin{array}{rl} & z_{ija}\leq x_{ia}\;\forall \;i\in I,j\in I\setminus \left\{i\right\},a\in A, \end{array}$$

(10) $$\begin{array}{rl} & z_{ija}\leq x_{ja}\;\forall \;i\in I,j\in I\setminus \left\{i\right\},a\in A, \end{array}$$

(11) $$\begin{array}{rl} & z_{ija}\geq x_{ia}+x_{ja}-1\;\forall \;i\in I,j\in I\setminus \left\{i\right\},a\in A, \end{array}$$

(12) $$\begin{array}{rl} & x_{ia}\in \left\{0,1\right\}\;\forall \;i\in I,a\in A, \end{array}$$

(13) $$\begin{array}{rl} & y_{iat},z_{ija}\in \left\{0,1\right\}\;\forall \;i\in I,a\in A,t\in T, \end{array}$$

(14) $$\begin{array}{rl} & q_{it}\geq 0\;\forall \;i\in I,t\in T, \end{array}$$

(15) $$\begin{array}{rl} & I_{it}\geq 0\;\forall \;i\in I,t\in T. \end{array}$$

The objective function (1) minimises setup, holding, and total unit cost, while taking cost synergies among items into account. These cost synergies depend on decision variable $z_{ija}$, indicating whether both, items *i* and *j*, are assigned to agent *a*. Only if two or more items *i* are assigned to an agent *a*, he or she can profit from cost synergies. Thus, we are facing a non-linear model. Cost synergies depend on $\omega_{ija}$, ${\bar{\omega }}_{ija}$, and ${\tilde{\omega }}_{ija}$, which give the factor by which setup cost $s_{ja}$, holding cost $h_{ja}$, and unit cost $c_{ja}$ are reduced, respectively.

Inventory levels are determined in constraint (2), and beginning inventories are defined in constraint (3). Endogenous demands are calculated in (4). Note that endogenous demand, $d_{it}$, cannot occur for final products, while exogenous demand, $D_{it}$, typically occurs for final products but can also occur for other products. We connect production quantities to setups in constraint (5). Setups of items *i* can only be done by agents *a* being in charge of this item. This is ensured in constraint (6). In constraint (7) we set the assignments of compulsory items. All compulsory items have to be assigned to the respective agent. Constraint (8) guarantees that each concurrent item is assigned to exactly one agent. We connect decision variables $x_{ia}$ and $z_{ija}$ in constraints (9)–(11). Constraints (12)–(15) define domains of variables. Note that this model is highly non-linear because of the multiple products in the objective (1). Hence, it is mainly used for precisely stating the problem, while it is hardly useful for solving the problem.

Cost synergies occur if costs are subadditive, i.e. the total cost for a set of items are less than the sum of all individual cost. This might be due to several reasons, like joint replenishment, setup, storing or transportation (see Section 1). Since we assume that producers are not willing to reveal sensitive information (e.g. costs like $s_{ja}$, $h_{ja}$, or $c_{ja}$), we cannot make use of a centralised solution approach, where a decision maker has access to the data required to solve the proposed mathematical model. Thus, we develop a decentralised approach, where a trading mechanism is used to indirectly share information of producers' preferences without forcing them to reveal individual item costs ($s_{ja}$, $h_{ja}$, and $c_{ja}$). This method can be used if agents are interested in collaborating in order to approximate a globally optimised solution. As an alternative approach, we design a myopic upstream planning procedure, where the agents do not have to interact with a central authority. Both decentralised approaches are assessed in comparison to centrally planned solutions, where the central authority is assumed to have full access to all individual costs.

While already the multi-level uncapacitated lot-sizing problem (Yelle 1979) is NP-hard for general product structures (Arkin, Joneja, and Roundy 1989), additional complexity is added by (i) having flexibility in the assignment of items to producers, and (ii) having costs that depend on this assignment. Note that if there are *n* concurrent items, we have $2^{n}-1$ possibilities to package them into bundles. The given problem can be generalised to the classical multi-level uncapacitated lot-sizing problem, by fixing assignment of items to agents. This would imply that variables $x_{ia}$ and $z_{ija}$ are fixed.

## 4. Solution methods

The proposed solution methods for the newly introduced problem have to (i) assign items to agents taking cost synergies into account, and to (ii) find production plans for each product. First we present a myopic upstream approach, where on each stage items are assigned to agents, who do a local optimisation for each product individually. In this method it is assumed that no collaborative planning is performed, and the agents do not have to interact with a central authority. For the second approach we assume that a central authority is in charge of coordinating the agents' collaborative activities. While these agents are willing to setup jointly orchestrated plans, they are not willing to share critical information like their individual item costs.

### 4.1. Upstream approach

First we propose a myopic upstream planning approach. The assignments of products to agents as well as setup decisions are taken on each BOM level individually. On the upmost level, i.e. for the end products, each product is assigned to its cheapest producer. For calculating the cost for an item, each agent defines his or her individual and optimal production plan for each product. This is done by solving the single-level uncapacitated lot-sizing problem for each item *i* to optimality:

(16) $$\begin{array}{rl} & \min\sum_{t\in T}(s_{i}y_{it}+h_{i}I_{it}+c_{i}q_{it}), \end{array}$$

(17) $$\begin{array}{rl} & I_{it}=I_{it-1}+q_{it}-d_{it}-D_{it}\;\forall \;t\in 1,\ldots ,\tau , \end{array}$$

(18) $$\begin{array}{rl} & I_{i0}=0, \end{array}$$

(19) $$\begin{array}{rl} & d_{it}=\sum_{j\in \Gamma (i)}p_{ij}Q_{jt}\;\forall \;t\in T, \end{array}$$

(20) $$\begin{array}{rl} & q_{it}\leq y_{it}M\;a\in A,t\in T, \end{array}$$

(21) $$\begin{array}{rl} & y_{it}\in \left\{0,1\right\}\;\forall \;t\in T, \end{array}$$

(22) $$\begin{array}{rl} & x_{it}\geq 0\;\forall \;t\in T, \end{array}$$

(23) $$\begin{array}{rl} & I_{it}\geq 0\;\forall \;t\in T, \end{array}$$

where we use the same notation as for the multi-level problem, omitting index *a* for the assignment of items to agents. In the single-level problem, the term for the unit cost ($c_{i}q_{it}$) is a constant. However, since we want to relate this formulation to the model given in (1)–(15), we keep the unit costs in the objective function. $Q_{jt}$ is the given quantity of item *j* being produced in period *t*. This is the result of the local optimisation on the previous BOM level.

On the next BOM level, each item is again assigned to its cheapest producer. For identifying the cheapest producer, each agent calculates the total cost according to (16), and reduces it by his or her cost synergies as in the centralised model. The demand is determined by production plans of the next higher BOM level, which is the reason that the procedure has to start with the end products. Total individual costs on each level are used to identify the cheapest producer of an item. We assume that agents have cost synergies if they already produce a product on the same level or if they produce a direct successor of this product (see Section 5).

This planning approach reflects a situation, where agents are not willing to cooperate. It can be seen as a best price allocation procedure, i.e. a producer procures components from his or her cheapest supplier. For this, offers from all potential suppliers are gathered and the cheapest one gets the offer. The cheapest producer is always chosen in a myopic and selfish way, i.e. synergies with lower levels are disregarded. We go through the BOM once, and do not perform backward iterations.

### 4.2. Decentralised 3-phase solution approach

In this second approach we assume that agents are willing to participate in a collaborative system, where they have to interact with a central authority in order to minimise total cost, which can be determined by objective function (1). The type of interaction depends on the phase of the solution approach as it is described below. However, we still assume that agents are not willing to reveal detailed information on their individual setup and holding cost.

The solution approach consists of three phases. The first one is used to find good production plans, while the second one assigns items to agents. The third phase uses some local optimisation. In phases 1 and 2, the agents are offered production plans, which they have to evaluate. These production plans are generated by a GA-based mechanism. The whole mechanism is assumed to be steered by a central authority.

#### 4.2.1. Phase 1: production plans

In this phase the mediator uses a GA to find good production plans. The initial population is generated randomly using 50% probability for entering 0 or 1. When generating plans, we, however, consider the following proposition given in Afentakis and Gavish (1986), which is a property that the optimal solution must fulfil: if there is a setup in period *t*, i.e. $y_{it}>0$, all successors of *i* (Γ(i)) also have to be setup in *t*. Furthermore, we have to ensure that for each item there is a setup in the first period where $d_{it}>0$. By this we guarantee feasibility of plans.

A GA operates with a set of solutions (called population), which is manipulated, by evolution-inspired methods like inheritance, crossover, mutation etc., to produce new populations with better solutions. GAs have been used to solve various optimisation problems; an application to the multi-level unconstrained lot-sizing problem is given by Homberger (2008). In what follows we describe our basic GA components, which are encoding and decoding of population individuals, inheritance operators, and fitness evaluation.

**Figure 2.**
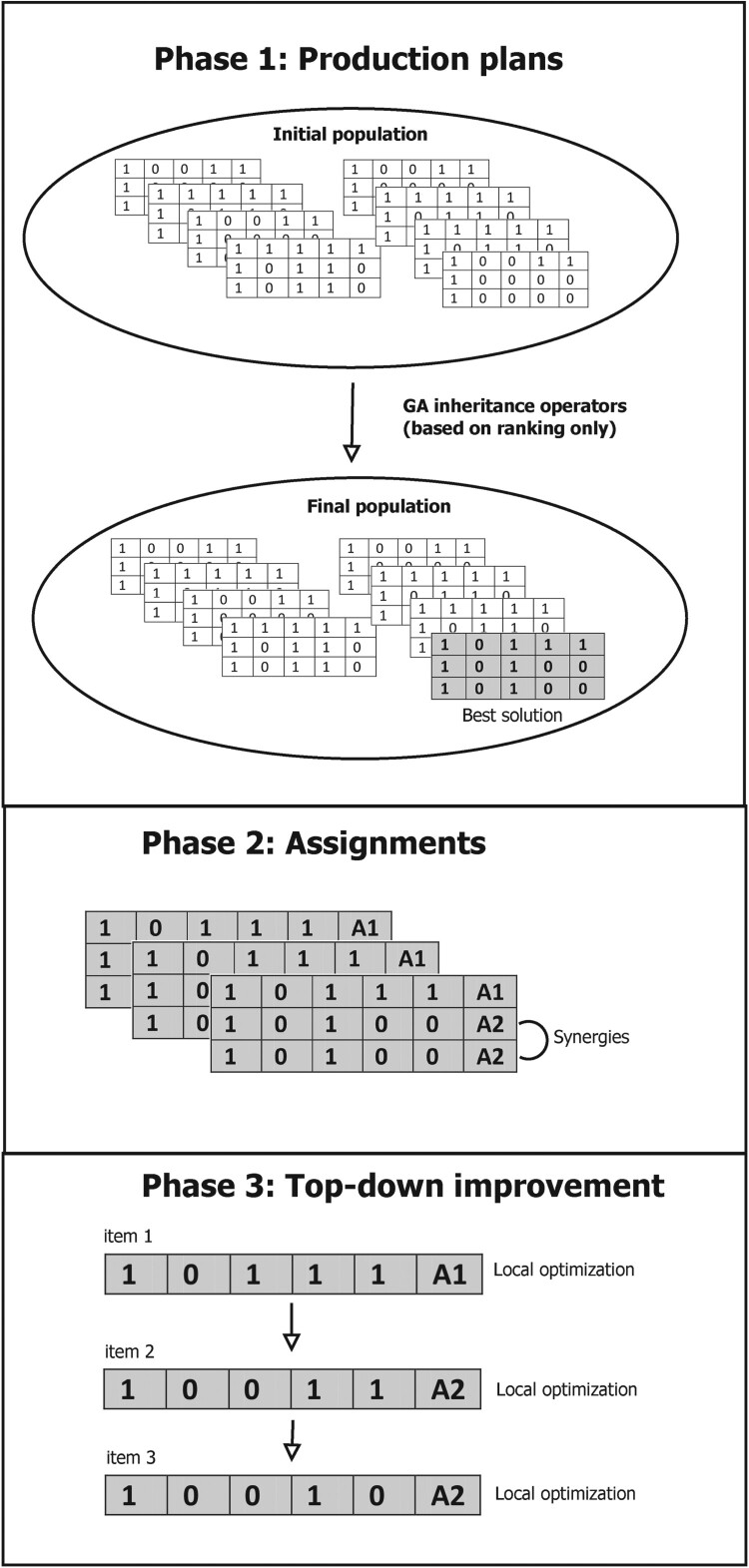
Illustration of the decentralised solution approach for a problem with three items and five periods. For a better visualisation we assume only one item per BOM level.

In the proposed mechanism, an individual is a production plan for all items in the product structure, i.e. a binary matrix with *n* rows and *τ* columns, where *τ* is the number of periods (see upmost part of Figure 2). Populations of individuals are offered to the agents, who are then asked to give their evaluations. Since we assume that agents are not willing to reveal their individual cost, agents have to rank all individuals within the population. If the agents would have to provide the cost for each production plan, the mediator could compute fixed cost and holding cost for all products of the agent by solving a system of linear equations after a finite number of iterations. Hence, we do not require the agents to reveal their cost but just a ranking of alternative solutions and average cost. Thus, if a population consist of *ε* individuals, i.e. production plans for all items, each agent *a* reports the rank $r_{pa}$ of each individual *p*, where the cheapest plan has rank 1, and the most expensive one rank *ε*^1^. For this ranking, each agent assumes that he or she produces all of his or her potential items. Note that a plan with no concurrent products can be more expensive than a plan with many concurrent products, if the plan includes expensive lot-sizing decisions for the compulsory products. Moreover, additional products lead to cost synergies. Hence, it can be beneficial to produce more products.

We calculate the fitness $f_{p}$ of an individual *p* by the following formula^2^:

(24) $$f_{p}=\frac{1}{\sum_{a\in A}r_{pa}^{2}}.$$

A new population is generated using standard operators: (i) elitism, (ii) crossover, and (iii) mutation.

*Elitism.* If a new population is created, the fittest candidates of the previous generation are carried over without any change.

*Crossover.* Individuals for crossover are chosen by a roulette wheel based on their fitness rank. We apply a standard uniform crossover (Michalewicz 1996) to add new individuals to a population. This means that with equal probability the entry in the binary matrix of a new individual is filled with the value of parent 1 or parent 2.

*Mutation.* An entry in a newly generated individual is mutated with probability 1/nτ (Homberger 2008).

Using these inheritance operators, new individuals are generated until the fixed population size is reached. Note that the GA is only used by the central authority. She produces new plans and offers them to agents. For the GA only rankings are used, no prices have to be revealed.

#### 4.2.2. Phase 2: assigning items to agents

In this phase, we start by selecting the ϑ best plans, evaluated with objective function (24) out of the final population of phase 1. For each of these plans we randomly generate *θ* possible assignments of items to agents. We evaluate these plans by calculating synergy coefficient $syn_{a}$ for each agent *a*. This coefficient sums up the (i) number of items an agent has on the same BOM level ($\lambda_{a}$) and (ii) the number of directly linked items that are both assigned to the same agent ($\nu_{a}$):

(25) $$syn_{a}=\lambda_{a}+\nu_{a}.$$

We select *κ* plans having the highest synergy coefficients and offer them to the agents. At this point the agents have to give their total cost for all of their items including synergies. Note that agents do not have to reveal their individual costs for single items. However, if *κ* is very high compared to the number of items being assigned to the agents, it might be possible to derive information on individual costs. This is of course hindered by the fact that agents report total costs including potentially non-linear synergy effects among their items. In our computational study we show that *κ* can be set to a very low number, such that no conclusions can be drawn. Note that *κ* should be small enough such that the mediator cannot derive the agents' cost structure from the information given. The plan with the minimum total cost (summed up over all agents) is the final production and assignment plan. This production plan is the starting point for the final optimisation, i.e. Phase 3.

#### 4.2.3. Phase 3: upstream optimisation

After having finished the assignment of items to agents, each agent tries to improve his or her individual cost by doing a local optimisation for each of his or her items. This phase is similar to the pure upstream approach (see Section 4.1). The agent producing the finished products report their locally optimised production plans to those on the next BOM level and so on. For the local optimisation each agent solves the single-level problem given in (16)–(23) to optimality and passes on his or her production plan for this product to the next level. Starting with the end products we optimise level-by-level with no backward iterations.

The procedure of the proposed 3-phase solution approach is illustrated in Figure 2. For better visualisation we assume that there are only three products being produced on three levels.

Dealing with collaborative planning brings up game theoretical aspects as well. In particular, the question arises whether agents are self-interested and opportunistic supply chain members, or whether they are willing to accept production plans and assignments that are favourable for the supply chain as a whole. This further leads to the question whether they are willing to act truthfully, irrespective of their individual interest (Stadtler 2009).

It would of course be desirable to have an incentive compatible mechanism, which implies that there are no incentives for participants to act untruthfully. According to the revelation principle (Myerson 1979), it is always possible for any mechanism to find an equivalent incentive compatible (IC) mechanism, that generates the same equilibrium allocation. Therefore, IC does not restrict the possibility to find an adequate mechanism for a problem but still it is a property that a meaningful mechanism needs to fulfil. Second price auctions (Vickrey 1961) or more general Vickrey–Clarke–Groves auctions Vickrey (1961), Clarke (1971) and Groves (1973) are known to be incentive compatible. However, due to the interdependencies among production plans, a straightforward auction process is not applicable for the investigated problem. The auctioneer cannot offer bundles of items separately but has to offer production plans for the whole product structure. Thus, the proposed mechanism can be seen as a combinatorial auction, where the bundling and the bidding process have to be done jointly but there is no individual price building phase. Besides this, second price auctions are impractical and rarely used in practice (Pekeč and Rothkopf 2003). While they are incentive compatible for the auction's bidding process, they might still be subject to several kinds of cheating, which in our case might be the information which items an agent is able to produce. Pekeč and Rothkopf (2003) claim that they are unsustainable in realistic dynamic environments in which the revelation of the bidder's values has consequences beyond the auction.

It has been shown by Gansterer, Hartl, and Vetschera (2018) that incentive compatible mechanisms in transportation auctions ex post lead to violations of other desirable properties like individual rationality of participants. Moreover, double or two-sided auctions, where participants are both buyers and sellers at the same time, are among the most prevalent forms of economic transactions (Kojima and Yamashita 2017). In our case, complexity is increased by the fact that all participants can be sellers and buyers at the same time, and that traded items have synergies among them. To the best of our knowledge, there is no mechanism available in the literature that realises all desirable properties for such markets. It has been shown in Gansterer and Hartl (2018a) that profitable strategic behaviour in transportation auctions is not easy to find. The outcome of strategic bidding is hard to predict and a profitable cheating strategy is not straight forward and potentially does not even exist.

For all these reason we align with Landeros and Monczka (1989) and assume that a supplier–buyer partnership is based on a trustworthy commitment of future behaviour. Thus, the assumption of non-strategic behaviour may not be unrealistic (Stadtler 2009).

However, for some of the agents, the joint plan might imply that they are worse off compared to their locally optimised solutions of their compulsory items. This can imply that a compensation scheme has to be setup. A scheme based on the well-known Shapley value is proposed by Buer, Ziebuhr, and Kopfer (2015). It is applied to the distributed lot-sizing problem with rivalling agents, and is also applicable to the problem with cost synergies. We therefore do not focus on compensation schemes in our study.

### 4.3. Benchmark: centralised planning

**Figure 3.**
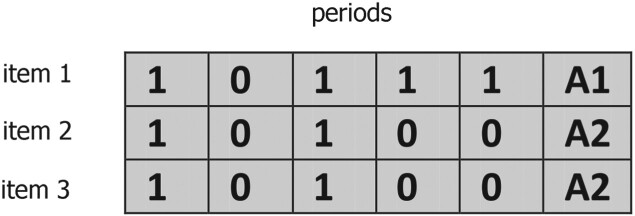
Illustration of an individual in the centralised GA for a problem with three items, five periods, and three agents.

In order to assess the performance of the proposed solution approaches, we also consider a central planner having full information on all individual costs. Hence, the central planner solves models (1)–(15). The centralised solutions for the problem given in Section 3 are generated using an adapted version of the GA proposed by Homberger (2008). While Homberger (2008) only tackles the multi-level unconstrained lot-sizing problem, we have to take the assignment of items to agents into account. Thus, we extend the proposed binary solution representation by this assignment. An individual in the centralised GA, assigns each product to an agent, and gives a production plan (0 or 1) in each period. An example for an individual for a problem with three items, five periods, and three agents is illustrated in Figure 3.

Since in the centralised approach, we assume that agents are willing to reveal their individual cost, we can calculate the fitness $f_{p}$ of an individual *p* by the following formula:

(26) $$f_{p}=\frac{1}{\sum_{a\in A}\phi_{pa}},$$

where $\phi_{pa}$ are the total costs of agent *a* when producing according to production plan *p*.

Similar to the GA used in phase 1 of the decentralised planning approach (see Section 4.2) we use (i) elitism, (ii) crossover, and (iii) mutation as inheritance operators.

*Crossover.* Individuals for crossover are chosen by a roulette wheel based on their fitness rank. Again, we apply a uniform crossover operator (Michalewicz 1996), which for the centralised approach has to be applied to both the distribution of setups in the production plan as well as the assignment of items to agents. Thus, with equal probability the entry in the binary matrix of a new individual as well as the agent per item is filled with the value of parent 1 or parent 2.

*Mutation.* An entry in the production plan of a newly generated individual is mutated with probability 1/nτ (Homberger 2008). The assignment in a newly generated individual is mutated with probability 1/2n.

Using these inheritance operators, new individuals are generated until the fixed population size is reached. The initial population is generated randomly using 50% probability for entering 0 or 1, and a randomly selected agent. Again we generate plans by taking the property of optimal production plans proposed by Afentakis and Gavish (1986) into account (see Section 4.2).

## 5. Computational study

For our computational study we use the publicly available test instances proposed by Buer, Ziebuhr, and Kopfer (2015) for the distributed multi-level lot-sizing problem with rivalling agents. There are two groups of instances: (1) small ones with 5 items being produced in 12 periods, (2) larger ones, where up to 50 items have to be produced in up to 24 periods. In each group there can be two or five agents in charge of producing the items. The first group is denoted as *s1–s96* and the second one as *m1–m40* by Buer, Ziebuhr, and Kopfer (2015).

We adapt these test instances by including cost synergies among items. We distinguish between cost synergies for items *i* and *j*, where *j* is a direct successor of *i*, i.e. j∈Γ(i), and cost synergies for items *i* and *j* being on the same or on a very close BOM level.

All experiments are coded in C++ and executed single threaded on an Intel Core i5-3570 3.4 GHz computer with an average runtime of 15 seconds per instance.

### 5.1. Cost synergies due to predecessors

Let us assume that *j* is a direct successor of *i*, i.e. j∈Γ(i). If *i* and *j* are assigned to the same agent, this agent *a* benefits from an agent specific cost synergy $\xi_{a}^{pred}$, which is randomly chosen between 0.8 and 0.9. We reduce setup and holding cost of *i* by multiplying them with $\xi_{a}^{pred}$:

(27) $$\omega_{ija}={\bar{\omega }}_{ija}=1-\xi_{a}^{pred}\;\forall \;j\in \Gamma (i).$$

#### 5.1.1. Cost synergies due to same or close BOM level

Let us define $l_{i}$ as the level of item *i* according to the product structure. If *i* and *j* are on the same level (i.e. $l_{i}=l_{j}$), we reduce the cost of *i* and *j* by multiplying both of them with the agent specific synergy factor $\xi_{a}^{level}$, which is randomly set between 0.8 and 0.9. If *i* is on level $l_{i}$ and *j* on level $l_{i}-1$, $\omega_{ija}$ is reduced by 50%. We do not assume cost synergies, if *j* is on level *l*−2 or more:

(28) $$\begin{array}{rl} \omega_{ija} & =\omega_{jia}={\bar{\omega }}_{ija}={\bar{\omega }}_{jia}=1-\xi_{a}^{level}\;\forall \;i,j:l_{i}=l_{j}, \end{array}$$

(29) $$\begin{array}{rl} \omega_{ija} & =\omega_{jia}={\bar{\omega }}_{ija}={\bar{\omega }}_{jia}=\frac{1-\xi_{a}^{level}}{2}\;\forall \;i,j:\left|l_{i}-l_{j}\right|=1. \end{array}$$

If *i* is on BOM level $l_{i}$ and *j* on level $l_{i}-1$, and *j* is a direct successor of *i*, i.e. j∈Γ(i), both synergies ($\xi_{a}^{pred}$ and $\xi_{a}^{level}$) apply:

(30) $$\omega_{ija}={\bar{\omega }}_{ija}=(1-\xi_{a}^{pred})\frac{1-\xi_{a}^{level}}{2}\;\forall \;i,j:j\in \Gamma (i)\wedge \left|l_{i}-l_{j}\right|=1.$$

In our computational study, we do not take unit cost into account since we want to analyse the effects of synergies on setup and holding costs. Thus, $c_{ia}$ in (1) is set to a fixed value ${\bar{c}}_{i}$, while all ${\tilde{\omega }}_{ija}$ are set to 0. This is in line with Buer, Ziebuhr, and Kopfer (2015).

In both, the centralised and the decentralised GA, we assume an elite size of 5 and population size of 500. The algorithm is stopped after generating 200 populations. In the 3-phase approach (see Section 4.2), *κ*, i.e. the number of proposed assignments, is set to 50, while ϑ and *θ* are set to 10 and 10,000, respectively.

### 5.2. Assessment of centralised planning approach without cost synergies

In order to assess the performance of the centralised GA, we first compare it to the results presented in Buer, Ziebuhr, and Kopfer (2015) for the special case without synergy effects. It should be noted, that Buer, Ziebuhr, and Kopfer (2015) propose a negotiation approach, where a fully informed central planner does not exist. However, at some point the agents have to reveal their individual costs for single items. A summary of the results is given in Table 1. It should be noted that in these experiments we do not consider cost synergies. It is just used to benchmark the results found by the proposed alternative planning approaches.

**Table 1.** The special case with no synergies: average percentage cost reduction (−) or increase (positive numbers) of the centralised planning approach (CEN), the upstream approach (UP), and the decentralised mechanism (DEC) compared to the decentralised negotiation approach by Buer, Ziebuhr, and Kopfer (2015).

				Gap		
Instances	Items	Agents	Periods	CEN	UP	DEC
s	5	2	12	−0.61%	−12.7%	1.6%
s	5	5	12	−2.8%	7.8%	−0.8%
Average				−1.7%	−2.4%	0.4%
m	40–50	2	12	0.0%	7.5%	3.4%
m	40–50	2	24	−1.2%	5.8%	4.8%
m	40–50	5	12	−8.7%	−0.8%	−1.8%
m	40–50	5	24	−10.8%	−5.4%	−4.8%
Average				−5.2%	1.8%	0.4%
Total average				−3.4%	−0.3%	0.4%

The results show that the CEN, which is the centralised GA presented in Section 4.3 is able to improve the best known solutions of almost all sets of test instances. In particular, instances *m* with 5 agents competing on up to 50 products in 24 periods have a huge solution space. For these instances, CEN can improve the best known solutions by more than 10%. UP, the myopic upstream approach (see Section 4.1), shows a very volatile performance. On average there is a slight improvement compared to Buer, Ziebuhr, and Kopfer (2015). However, there are some instance sets, where the results are more than 7% worse. It should be noted, that in the UP approach, items are assigned to the cheapest agent at each stage. Thus, agents have to reveal parts of their individual item costs. The decentralised mechanism (DEC), where agents do not have to reveal their item costs (see Section 4.2), comes very close to the results of the negotiation approach of Buer, Ziebuhr, and Kopfer (2015). In particular for instance sets with 5 rivalling agents, the DEC can improve the solutions of Buer, Ziebuhr, and Kopfer (2015). It should be noted that in these experiments there are no cost synergies considered. Cost synergies significantly increase complexity. This will be investigated in the next subsection.

### 5.3. Assessment of solution approaches for problems with cost synergies

In this main part of our computational study, we assess the performance of the two proposed methods for the problems with cost synergies. In Table 2 we report the percentage deviation of the upstream approach as well as those of the decentralised GA compared against the best known solutions generated by the centralised GA. Detailed results are available in Tables 3–5 in the Appendix.

**Table 2.** The general case with synergies: average percentage cost reduction of the upstream (UP) approach and the decentralised mechanism (DEC) compared to the centralised solution.

				Gap	
Instances	Items	Agents	Periods	UP	DEC
s	5	2	12	13.4%	4.1%
s	5	5	12	11.3%	1.6%
Average				12.3%	2.8%
m	40–50	2	12	8.5%	4.6%
m	40–50	2	24	9.4%	4.7%
m	40–50	5	12	15.0%	7.4%
m	40–50	5	24	12.8%	4.1%
Average				11.4%	5.2%
Total average				11.8%	4.0%

The results show a strong performance of the decentralised 3-phase planning approach DEC. On the small instances (upper part of Table 2) the deviation from the centralised solution is less than 3%. It should be emphasised that the centralised GA makes use of a fully informed central planner. Applying the upstream approach UP we observe an average deviation of 12.3%.

For the bigger instances (lower part of Table 2) the average deviation of DEC is 5.2%. We observe significantly higher deviations of 11.4% when using the upstream approach UP.

Our computational study shows that the proposed decentralised 3-phase mechanism is a highly attractive method for agents who want to collaborate but are not willing to share sensitive information on (i) individual item cost and (ii) potential cost synergies. While they only have to reveal the ranking of production plans, the collaboratively generated solution is on average only 4% worse than the solution found by a central decision maker having full information. For these experiments, *κ*, i.e. the number of proposed assignments (see Section 4.2), is set to 50. Thus, given the number of items and periods, it is mathematically not possible to derive the agents' cost structure from the proposed plans. Please note that the mediator has neither information on the agents' cost synergies nor on the models the agents use for calculating total costs.

Another interesting observation is that the upstream approach leads to very poor results if cost synergies are considered, while for some instance sets of the problem without synergies (Table 1) the upstream approach improves the solutions of the negotiation approach (Buer, Ziebuhr, and Kopfer 2015) and of the decentralised mechanism. This is due to the myopic procedure, where beneficial assignments of items to agents are not realised.

## 6. Conclusions

We introduced the collaborative multi-level lot-sizing problem with cost synergies. The problem is of particular relevance in the field of cloud manufacturing, where high modularisation and service-orientation force companies to pool operations. While the problem is a natural and highly practical extension of existing problems, it has not been presented in the literature so far. We provided the mathematical formulation and designed two decentralised solution approaches. We have shown that the proposed 3-phase planning approach, where a central authority estimates agents' preferences based on rankings of production plans, comes very close to the solutions generated by a central decision maker having full information. On average, the gap between centralised and decentralised solutions is only 4%, while in the decentralised one no critical information has to be shared.

We compared our method to a myopic upstream planning approach, where agents are not willing to collaborate. Although, each agent solves his or her individual lot-sizing problem to optimality, and also cost synergies are considered, this approach showed a loss in solution quality by up to 12.8%.

All planning approaches were compared against the best known solutions for the problem without cost synergies. The results showed that the proposed decentralised mechanism without information sharing performs very well for instance sets with a high number of rivalling agents. For these instances, the mechanism significantly improved the results found by a negotiation approach, where individual item costs have to be revealed. Another interesting observation was that the results of the myopic upstream approach without cost synergies were on average only 3.1% worse than the centralised solutions, while it showed very poor solution quality if cost synergies are taken into account. This strongly emphasises the strength of the proposed 3-phase mechanism since it is able to realise cost synergies.

Our study showed that, even if agents are not willing to share critical data, decentralised solution approaches can be used to reach high quality production plans. The proposed mechanism can be applied to other problem classes, where collaborative decision makers aim for good plans under incomplete information (e.g. collaborative replenishment or distribution).

## Notes

1 While in this study ranks are determined based on total production cost, ranks can also be based on, e.g. ratios between setup and production cost.

2 In a preliminary study we examined the following fitness function $f_{p}=1/\sum_{a\in A}r_{pa}$. However, this led to a loss in solution quality of up to 1.3%

## Appendix

### Appendix

**Table A1.** Total cost (best over 5 runs; including cost synergies) found using centralised (CEN), decentralised (DEC), and upstream (UP) approach for small instances (s01–s45) and 2 or 5 agents.

Instances	2 agents			5 agents		
	CEN	DEC	UP	CEN	DEC	UP
s01	292.48	301.23	396.40	293.09	298.76	402.54
s02	451.51	472.52	532.98	532.99	560.98	649.82
s03	648.30	657.06	807.28	711.51	715.10	815.25
s04	484.86	487.60	521.15	858.75	887.85	971.72
s05	298.14	305.23	361.21	327.33	342.96	421.79
s06	456.98	474.73	529.42	562.41	564.59	688.59
s07	561.45	600.41	715.88	708.94	729.66	827.07
s08	608.89	647.36	681.29	873.02	891.76	949.79
s09	249.33	267.65	308.73	299.19	310.69	365.24
s10	478.67	493.96	559.29	500.05	502.69	589.12
s11	484.62	507.96	607.91	621.62	631.59	698.59
s12	454.70	458.75	583.26	834.54	834.54	884.86
s13	290.68	314.47	351.15	302.93	316.91	401.08
s14	478.58	526.71	563.64	531.74	538.02	619.36
s15	595.07	601.52	730.39	667.70	675.99	771.92
s16	498.11	498.11	624.15	797.06	804.83	846.94
s17	251.10	265.97	303.44	265.82	265.82	350.66
s18	401.98	431.72	466.26	435.06	445.66	540.46
s19	588.92	606.05	645.54	631.45	645.44	722.20
s20	570.65	570.65	697.44	768.10	786.60	809.03
s21	228.37	228.81	271.79	259.06	262.50	315.61
s22	374.49	385.51	419.99	415.34	420.30	503.63
s23	364.70	375.93	408.77	532.99	532.99	610.65
s24	366.09	373.18	380.00	676.98	687.25	761.59
s25	474.82	494.06	494.06	504.02	506.72	520.22
s26	591.46	622.12	634.60	674.63	678.98	718.10
s27	670.92	690.38	772.19	767.09	786.65	786.65
s28	725.76	736.92	882.45	897.79	908.71	967.51
s29	407.62	421.28	431.79	448.47	448.87	463.36
s30	569.02	578.16	578.16	679.28	687.06	687.06
s31	767.31	768.94	768.19	879.57	879.66	879.66
s32	768.67	771.22	777.81	958.81	986.19	970.87
s33	369.37	376.53	376.53	417.20	421.00	427.40
s34	497.23	501.08	513.20	620.51	620.51	625.82
s35	584.14	587.99	686.08	758.06	763.16	797.92
s36	681.70	688.11	688.11	922.89	948.08	956.66
s37	377.13	388.92	397.67	378.20	387.81	387.81
s38	547.13	559.31	559.31	643.65	644.45	648.82
s39	599.30	604.56	690.03	764.33	785.27	785.27
s40	709.37	712.72	783.01	914.60	928.96	928.96
s41	358.35	374.74	382.14	402.98	407.28	419.08
s42	526.59	534.15	534.15	577.20	579.25	601.30
s43	644.94	646.05	646.05	757.10	761.36	758.20
s44	664.12	664.12	742.75	816.88	826.15	865.39
s45	324.69	347.24	347.24	367.58	368.58	372.58

**Table A2.** Total cost (best over 5 runs; including cost synergies) found using centralised (CEN), decentralised (DEC), and upstream (UP) approach for small instances (s46–s96) and 2 or 5 agents.

Instances	2 agents			5 agents		
	CEN	DEC	UP	CEN	DEC	UP
s46	453.11	455.36	458.61	515.64	515.64	526.10
s47	502.92	505.17	584.10	647.30	648.64	648.64
s48	549.00	549.00	606.89	702.74	712.83	750.87
s49	454.93	544.13	634.24	507.74	532.08	691.82
s50	598.43	726.80	751.63	717.39	723.65	870.19
s51	530.85	557.99	628.68	760.46	781.78	925.90
s52	498.40	500.81	525.84	811.45	861.62	1023.66
s53	430.87	524.18	602.89	524.19	544.92	708.87
s54	618.27	732.19	740.44	625.01	625.01	902.41
s55	736.00	777.21	835.65	820.22	829.26	973.44
s56	769.59	806.64	925.17	842.93	901.62	1087.67
s57	428.52	549.14	565.10	471.68	488.31	625.79
s58	570.33	598.79	718.70	686.21	686.21	819.46
s59	541.59	545.54	586.11	787.08	789.79	883.72
s60	723.08	731.81	865.75	856.45	856.45	926.49
s61	450.93	502.52	585.97	503.28	525.74	673.06
s62	608.32	657.81	721.13	656.68	669.66	820.69
s63	579.31	606.56	636.53	850.90	875.89	958.52
s64	530.94	533.00	543.43	814.12	814.12	908.22
s65	385.53	481.73	526.45	456.88	463.39	592.08
s66	547.35	578.79	717.17	622.31	631.69	796.16
s67	645.48	659.24	737.20	714.10	729.48	834.81
s68	440.72	482.81	671.22	769.91	797.29	929.04
s69	386.35	386.35	501.11	424.64	432.22	539.01
s70	450.07	510.49	588.86	514.51	514.51	678.22
s71	466.80	466.80	534.82	573.44	573.44	745.03
s72	420.26	438.25	611.70	705.99	705.99	787.95
s73	885.65	910.56	937.65	1032.44	1043.61	1067.67
s74	1057.93	1094.97	1102.68	1185.30	1221.90	1277.60
s75	838.04	852.65	988.22	1143.41	1164.18	1164.18
s76	826.42	845.78	950.26	997.92	1019.47	1019.47
s77	980.32	989.58	1076.15	1078.20	1086.24	1170.08
s78	982.70	1047.25	1088.95	1279.72	1289.83	1380.10
s79	941.64	963.49	1062.06	1130.20	1150.04	1189.73
s80	865.37	876.00	1023.95	1090.07	1104.75	1110.85
s81	875.70	920.48	920.48	981.56	990.48	1054.26
s82	934.24	1013.75	1013.75	1028.01	1035.07	1059.81
s83	868.06	871.48	890.75	1002.08	1013.88	1069.07
s84	791.03	791.03	881.36	992.32	1016.05	1006.33
s85	886.34	931.39	932.82	1008.54	1008.84	1061.12
s86	904.57	919.61	939.08	1131.02	1138.52	1155.41
s87	860.65	869.24	961.25	1007.58	1007.58	1047.48
s88	775.06	783.66	950.18	997.24	1034.87	1072.31
s89	811.98	821.52	823.95	909.06	914.76	967.20
s90	839.15	839.15	865.90	979.53	1011.53	1059.70
s91	755.57	755.57	860.12	940.97	988.74	1020.34
s92	524.73	524.73	704.99	880.16	880.16	890.33
s93	692.54	697.86	779.77	742.18	757.41	790.29
s94	779.92	815.88	815.88	871.23	882.45	922.60
s95	679.35	685.16	746.71	754.26	777.06	777.06
s96	444.73	444.73	444.73	828.36	828.36	854.97

**Table A3.** Total cost (best over 5 runs; including cost synergies) found using centralised (CEN), decentralised (DEC), and upstream (UP) approach for larger instances and 2 or 5 agents.

Instances	2 agents			5 agents		
	CEN	DEC	UP	CEN	DEC	UP
m01	24,053.07	25,324.42	25,324.42	77,163.64	80,817.03174	84,593.13
m02	40,035.56	42,442.32	44,680.70	85,938.00	93,474.77294	101,445.78
m03	24,271.39	24,802.42	24,802.42	55,682.97	59,444.21075	63,436.85
m04	41,050.89	43,490.28	43,490.28	74,301.32	81,292.25042	88,109.88
m05	28,774.44	29,742.08	29,742.08	86,283.14	92,744.15741	107,662.52
m06	45,280.11	48,161.18	51,597.91	90,031.74	99,983.36971	103,423.90
m07	32,418.99	33,306.16	34,127.74	65,224.02	68,314.90568	67,969.12
m08	29,362.42	32,223.70	32,223.70	48,966.97	52,601.81516	60,901.99
m09	22,252.69	23,600.97	23,600.97	68,749.73	75,560.85141	78,490.76
m10	40,806.50	43,656.64	46,472.24	78,999.23	88,168.90581	88,903.80
m11	47,352.59	48,507.85	53,889.48	100,175.39	106,837.5683	108,834.84
m12	68,671.77	70,493.01	70,493.01	92,491.76	98,709.6028	103,768.85
m13	37,821.45	38,999.93	41,381.00	119,700.35	130,565.7428	148,544.19
m14	92,792.07	95,580.01	106,673.90	193,908.84	212,932.9523	217,392.36
m15	43,238.05	43,937.87	47,557.74	140,040.86	148,532.5707	164,400.46
m16	78,022.22	82,911.78	83,925.75	162,298.47	186,185.9579	188,548.81
m17	44,058.86	44,940.24	45,364.22	136,960.18	150,531.6046	162,007.80
m18	115,991.58	122,443.94	124,888.97	134,657.35	137,584.634	139,671.99
m19	57,927.26	59,185.57	63,339.84	169,569.00	180,746.436	201,006.67
m20	93,795.42	100,198.86	100,198.86	191,385.06	214,966.6488	225,330.63
m21	37,086.50	39,679.72	41,404.91	65,294.01	68,572.49204	71,846.76
m22	43,842.15	46,023.32	46,023.32	94,124.13	101,176.4362	100,811.70
m23	56,250.90	57,248.87	57,248.87	99,904.92	105,642.7085	124,771.07
m24	36,903.50	38,716.66	40,311.74	94,429.74	100,146.5114	100,953.18
m25	35,676.41	37,323.96	41,336.36	81,660.37	87,982.36071	102,363.39
m26	45,858.28	48,965.61	49,708.90	74,648.99	80,386.2355	86,093.30
m27	48,515.40	49,985.11	49,985.11	90,344.12	95,550.07838	97,915.06
m28	31,744.01	34,272.98	35,088.91	69,572.22	72,803.13422	88,549.87
m29	43,938.57	46,067.60	48,238.54	85,525.62	90,940.94914	94,363.91
m30	30,601.89	31,802.04	36,235.33	65,950.04	67,069.23433	72,801.81
m31	91,541.95	95,515.45	95,515.45	147,346.83	156,940.5789	178,986.50
m32	63,343.87	68,768.79	68,249.58	152,004.71	161,966.6732	170,810.50
m33	97,444.45	101,164.52	102,908.73	181,835.90	189,925.7012	196,631.65
m34	63,943.18	66,276.83	82,254.82	129,707.78	134,472.8598	148,260.41
m35	99,384.47	103,932.01	105,132.10	161,106.44	165,466.7204	181,345.98
m36	79,149.33	84,601.72	84,601.72	142,612.85	144,006.1565	144,456.47
m37	113,605.86	118,058.47	118,058.47	221,883.52	235,192.0156	259,121.35
m38	85,963.08	90,110.34	92,644.18	190,188.38	196,245.2845	202,544.23
m39	97,122.15	101,983.20	126,277.98	179,214.69	188,609.9436	191,365.13
m40	66,019.34	67,422.19	67,422.19	126,277.93	128,765.4536	136,226.14
